# Evaluation of efficiency and quality of the multi-disciplinary team handover process in a mother and baby inpatient setting

**DOI:** 10.1192/j.eurpsy.2021.1339

**Published:** 2021-08-13

**Authors:** A. Daud, B. Kesete, S. Sriranjan, E. Britton, C. Steel, J. Lewin

**Affiliations:** Coombe Wood Perinatal Mental Health Unit, Central and North West London NHS Foundation Trust, London, United Kingdom

**Keywords:** service evaluation, safety, handover, quality improvement

## Abstract

**Introduction:**

At Coombe Wood Mother and Baby unit (MBU) there are daily multi-disciplinary team (MDT) handover meetings and a weekly MDT ward round attended by 7-8 team members. There are concerns that the handover is too time consuming, utilising time which could be spent on other clinical duties, and concerns regarding the relevance of information that is handed over.

**Objectives:**

To perform a service evaluation to determine the efficiency and quality of MDT handover meetings in an MBU setting.

**Methods:**

Data was collected from September to October 2020. A checklist was designed listing information felt to be relevant to handover and contained the following data points – ‘current situation’, ‘mental health’, ‘level of observations’, ‘risk’, ‘physical health’, ‘baby care’, ‘baby supervision levels’ and ‘tasks and responsibilities’. The start and stop times of each MDT handover meeting were noted and a record was made as to whether these topics were discussed.

**Results:**

Mean meeting duration was 32.2 minutes (range: 13 – 45 minutes) and amounted to 2.68 hours spent in MDT handover over a 5-day working week. This equates to 21.4 person-hours (based on 8 staff) a week. 928 data points were generated. 50.7% (468) data points were recorded and commonly omitted data points were – ‘tasks and responsibilities’, ‘risk’, ‘level of observations’ and ‘physical health’. On all occasions, ‘current situation’, ‘mental health’ and ‘baby care’ were handed over.

**Conclusions:**

The results of this service evaluation provide compelling evidence for a wider improvement project. Involving MDT staff in designing interventions will make handover meetings more meaningful.

